# *Sanguisorba officinalis* L. Ameliorates Hepatic Steatosis and Fibrosis by Modulating Oxidative Stress, Fatty Acid Oxidation, and Gut Microbiota in CDAHFD-Induced Mice

**DOI:** 10.3390/nu15173779

**Published:** 2023-08-29

**Authors:** Yunseong Nam, Myungsuk Kim, Saruul Erdenebileg, Kwang Hyun Cha, Da Hye Ryu, Ho Youn Kim, Su Hyeon Lee, Je Hyeong Jung, Chu Won Nho

**Affiliations:** 1 Division of Bio-Medical Science and Technology, KIST School, University of Science and Technology (UST), Seoul 02792, Republic of Korea; nys5167@kist.re.kr (Y.N.); g-sstainer@kist.re.kr (M.K.); 619008@kist.re.kr (S.E.); chakh79@kist.re.kr (K.H.C.); hykim@kist.re.kr (H.Y.K.); 2 Smart Farm Research Center, Korea Institute of Science and Technology (KIST), Gangneung 25451, Republic of Korea; dahye0507@kist.re.kr (D.H.R.); leesh@kist.re.kr (S.H.L.); jhjung@kist.re.kr (J.H.J.); 3 Natural Product Research Center, Korea Institute of Science and Technology (KIST), Gangneung 25451, Republic of Korea; 4 Department of Convergence Medicine, Wonju College of Medicine, Yonsei University, Wonju 26493, Republic of Korea; 5 Natural Product Informatics Research Center, Korea Institute of Science and Technology (KIST), Gangneung 25451, Republic of Korea

**Keywords:** *Sanguisorba officinalis* L., steatosis, fibrosis, gut microbiota, choline-deficient, L-amino acid-defined, high-fat diet (CDAHFD)

## Abstract

Non-alcoholic fatty liver disease (NAFLD) is a leading cause of chronic liver diseases and encompasses non-alcoholic steatosis, steatohepatitis, and fibrosis. *Sanguisorba officinalis* L. (SO) roots have traditionally been used for their antioxidant properties and have beneficial effects on metabolic disorders, including diabetes and obesity. However, its effects on hepatic steatosis and fibrosis remain unclear. In this study, we explored the effects of a 95% ethanolic SO extract (SOEE) on NAFLD and fibrosis in vivo and in vitro. The SOEE was orally administered to C57BL/6J mice fed a choline-deficient, L-amino-acid-defined, high-fat diet for 10 weeks. The SOEE inhibited hepatic steatosis by modulating hepatic malondialdehyde levels and the expression of oxidative stress-associated genes, regulating fatty-acid-oxidation-related genes, and inhibiting the expression of genes that are responsible for fibrosis. The SOEE suppressed the deposition of extracellular matrix hydroxyproline and mRNA expression of fibrosis-associated genes. The SOEE decreased the expression of fibrosis-related genes in vitro by inhibiting SMAD2/3 phosphorylation. Furthermore, the SOEE restored the gut microbial diversity and modulated specific bacterial genera associated with NAFLD and fibrosis. This study suggests that SOEE might be the potential candidate for inhibiting hepatic steatosis and fibrosis by modulating oxidative stress, fatty acid oxidation, and gut microbiota composition.

## 1. Introduction

Non-alcoholic fatty liver disease (NAFLD) is a leading cause of chronic liver diseases, with an estimated worldwide prevalence of 32.4% [1]. Moreover, its prevalence has increased over time, from 25.5% in 2005 to 37.8% in 2016 [2]. NAFLD is characterized by the excessive accumulation of lipids in hepatocytes that was not due to alcohol consumption [3]. It is defined as fat deposition in the hepatocytes with a lipid content > 5% on biopsy [4]. NAFLD is a group of diseases that encompasses non-alcoholic fatty liver (NAFL) and non-alcoholic steatohepatitis (NASH), which is classified as a progressive NAFLD that can progress to fibrosis and cirrhosis, and in some cases, hepatocellular carcinoma (HCC). About 30–40% of NAFLD patients progress to NASH, and 30–45% of NASH patients progress to liver fibrosis, increasing the morbidity of cirrhosis and HCC [5]. Despite the increasing prevalence of NAFLD, no FDA-approved medications for NAFLD are available. 

Various studies on NAFLD, including those investigating NAFLD progression, drug efficacy, and associated molecular mechanisms, have been conducted using diet-induced animal models [6]. A common diet-induced model used in NAFLD studies is the methionine- and choline-deficient (MCD) diet [7,8,9]. An MCD diet induces NAFLD and fibrosis by inhibiting hepatic fatty acid oxidation and increasing circulatory very low-density lipoprotein (VLDL) levels [10]. However, MCD diet models induce cirrhotic weight loss and cachexia, which are not the hallmarks of NAFLD in humans [11]. A high-fat, high-fructose, high-cholesterol (HFFC) diet results in steatosis, NASH, and fibrosis. However, an HFFC diet requires at least six months of intake to induce hepatic steatosis and fibrosis [12]. A choline-deficient, L-amino-acid-defined (CDAA) diet-induced model overcomes weight loss challenges and effectively develops NAFLD and fibrosis [13]. In addition, a choline-deficient, L-amino-acid-defined, high-fat diet (CDAHFD) leads to liver fibrosis progression within a short period in a diet-induced NAFLD mouse model, which is potentially useful for mimicking human NAFLD [6,9]. Previous studies used CDAHFD to induce NAFLD and fibrosis and confirmed the efficacy of drugs and natural products [14,15].

*Sanguisorba officinalis* L. (SO) is a perennial plant found in Asia. The root of SO has been established as a traditional medicinal plant in Korea to treat hemostasis, burns, and inflammation [16,17]. Moreover, various studies reported the bioactivities of SO roots, including anti-inflammatory, antioxidant, and anti-obesity activities [18,19,20]. Among the compounds derived from the roots of SO are triterpenoids and phenolic acids, such as gallic acid, catechin, ziyuglycoside I (ZG1), and ziyuglycoside II (ZG2) [21,22]. Specifically, ZG1 and ZG2, which exhibit anti-inflammatory and anticancer effects, were exclusively found in the roots of SO [22,23,24]. Despite the physiological activity of the SO root and its derived compounds, their effects on hepatic steatosis and fibrosis have not yet been investigated. In this study, the bioactive effects of SO root 95% ethanol extract (SOEE) were investigated to determine its potential therapeutic effects against hepatic steatosis and fibrosis in CDAHFD-fed mice.

## 2. Materials and Methods

An extended methods section is available in the Supplementary Materials.

### 2.1. Plant Materials and Extraction

Dried roots of SO were collected from China in November 2020 and purchased from a commercial market (Samhong, Gyunggi-do, Republic of Korea) in South Korea. Plant identification was confirmed by Professor Dae Sik Jang of Kyung Hee University (Seoul, Republic of Korea). The SO roots were dried and crushed into a powder. Powdered SO roots (50 g) were extracted with 500 mL of 95% ethanol for 72 h at room temperature on an orbital shaker and filtered through filter paper (GE Healthcare, Chicago, IL, USA). A rotary evaporator was used to evaporate the solvent in the SOEE under reduced pressure (Buchi, Flawil, Switzerland) to eliminate the solvent. The chromatogram of SOEE and standard compounds (ZG1, ZG2, and gallic acid) (Figure S1), chemical analysis information (Tables S1–S3), and quantification of each compound (ZG1, ZG2, and gallic acid) (Table S4) are shown in the Supplementary Materials.

### 2.2. Cell Culture 

The LX-2 cell line was obtained from the American Type Culture Collection (Manassas, VA, USA). The cells were cultured using Dulbecco’s Modified Essential Medium (Welgene, Gyeongsan-si, Gyeongsangbuk-do, Republic of Korea) supplemented with heat-inactivated fetal bovine serum (10%) (Gibco, Grand Island, NY, USA) and penicillin-streptomycin (1%) (Hyclone, Logan, UT, USA) and incubated at 37 °C in 5% CO_2_. 

### 2.3. Animal Experiment

Four-week-old C57BL/6J male mice were purchased from Daehan Biolink Inc. (Eumseong, Chungcheongbuk-do, Republic of Korea) and acclimated for 2 weeks. The mice were maintained in a controlled environment facility (23 ± 0.5 °C, 60% humidity) with a 12/12 h light/dark cycle. After adaptation, the mice were divided into five feeding groups: control diet group (control, *n* = 10), CDAHFD (A06071302, Research Diets Inc., New Brunswick, NJ, USA) group (CDAHFD group, *n* = 10), CDAHFD treated with 15 mg/kg/day obeticholic acid (Medchemexpress, Princeton, NJ, USA) (15 mg/kg/day) (OCA group, *n* = 10), CDAHFD treated with the SOEE (25 mg/kg/day) (SO25 group, *n* = 10), and CDAHFD treated with the SOEE (100 mg/kg/day) (SO100 group, *n* = 10). The SOEE dosage was determined by referring to previous reports on metabolic diseases [20]. Doses of 25 and 100 mg/kg/day of SOEE in mice are equivalent to 121.6 and 486.5 mg, respectively, of SOEE in humans (60 kg) [25]. The OCA and SOEE were dissolved in 0.5% carboxymethyl cellulose solution. Each treatment was administered daily via oral gavage for 10 weeks. One day before the mice were sacrificed, feces were collected from each mouse. After 10 weeks, the mice were sacrificed and plasma and liver tissues were collected and stored at −80 °C for biological analysis. An overview of the experimental design is shown in Figure S2. 

### 2.4. Biochemical Analysis

The plasma levels of alanine aminotransferase (ALT) (Cat. E-BC-K235-S), aspartate aminotransferase (AST) (Cat. E-BC-K236-M), TG (Cat. E-BC-K238), and total cholesterol (TC) (Cat. E-BC-K109-M) were quantified using kits from Elabscience (Houston, TX, USA). TG, TC, malondialdehyde (MDA) (Cat. E-BC-K025-M), and hydroxyproline (Cat. E-BC-K062-S) levels in the liver tissues were measured using kits from Elabscience (USA). All experiments were performed according to the manufacturer’s instructions.

### 2.5. Histological Examination

The right lobe of the liver from each mouse was collected, fixed, and stained for histological examination. A detailed description of the methods is given in the Supplementary Materials. 

To evaluate the NAFLD activity score (NAS), the scores of various histological features, such as steatosis, ballooning, and inflammation, were computed using a pre-trained convolutional neural network (CNN) [26]. Briefly, high-magnification image tiles in two dimensions (299 × 299 px^2^) were prepared using a Zeiss AxioCam MRc5 (Carl Zeiss, Jena, Germany) microscope with 125× magnification and 0.44 µm/px of pixel resolution under bright field illumination [26]. Image tiles were quantified and described using an automated “Kleiner score” [27] of steatosis (0–3), ballooning (0–2), and inflammation (0–3) and then summed up to give the NAS [26]. NAS was evaluated based on H&E-stained liver sections using a CNN.

### 2.6. Quantitative Reverse Transcription–Polymerase Chain Reaction (qRT-PCR)

Total RNA was extracted from liver tissue and LX-2 cells and complementary DNA (cDNA) was synthesized. qRT-PCR was performed using gene-specific primers (Table 1). Detailed information is described in the Supplementary Materials.

### 2.7. Western Blotting

To determine the protein expression levels, cell lysates were harvested and centrifuged to obtain proteins. The primary antibodies used were anti-rabbit SMAD2/3, phosphor-SMAD2/3, and glyceraldehyde-3-phosphate dehydrogenase (GAPDH). Goat anti-rabbit IgG antibody (horseradish peroxidase (HRP)-conjugated) was used as the secondary antibody. Detailed information and methods are described in the Supplementary Materials.

### 2.8. 16S rRNA Gene Sequencing of the Bacterial Community in Feces

One day before sacrifice, fecal samples were collected, homogenized via mechanical lysis using bead beating, and DNA was extracted. To amplify the V3–V4 hypervariable region of the 16S rRNA gene, PCR was performed using the universal primer set 341F and barcoded 806R. Samples were sequenced on a MiSeq platform and raw sequencing data were analyzed using the QIIME2-DADA2 pipeline [28,29]. Detailed information and methods are described in the Supplementary Materials.

### 2.9. Statistical Analysis

All results are presented as the mean ± standard error (SEM). Statistical differences between groups were evaluated using one-way analysis of variance (ANOVA) and Tukey’s post hoc multiple comparison test using R software (v.4.1.2.). A *p*-value < 0.05 was considered statistically significant. Spearman’s correlation was used to correlate the phenotypes and abundances of the microbial taxa. The *p*-values were adjusted using the Benjamini–Hochberg (BH) false discovery rate (FDR) procedure [30], and the correlation coefficients and adjusted *p*-value were visualized using the “heatmap” package [31].

## 3. Results

### 3.1. Effects of SOEE on Plasma Lipid Profiling and Liver Injury Markers in CDAHFD-Fed Mice

To explore the effects of the SOEE on NAFLD and fibrosis in vivo, male mice were fed a CDAHFD for 10 weeks to develop NAFLD and fibrosis and then orally administered 25 or 100 mg/kg/day of SOEE. OCA (15 mg/kg/day, farnesoid X Receptor agonist) was used as the positive control. First, the circulating levels of ALT and AST, which are liver injury markers, were examined to determine the effects of the SOEE on the liver. The levels of ALT and AST were significantly elevated in the CDAHFD group; however, the SOEE treatment did not significantly reduce these levels (Table 2). In contrast, the OCA administration increased the levels of these liver injury markers.

The TG levels in the plasma increased in the CDAHFD group, whereas it was significantly decreased by the OCA and SOEE administration (*p* < 0.05) (Table 2). However, the TC levels were lower in the CDAHFD group than in the control group, and the administration of OCA and SOEE did not result in significant alterations.

### 3.2. Effect of SOEE on NAFLD-Related Traits in CDAHFD-Fed Mice

**Figure 1:**
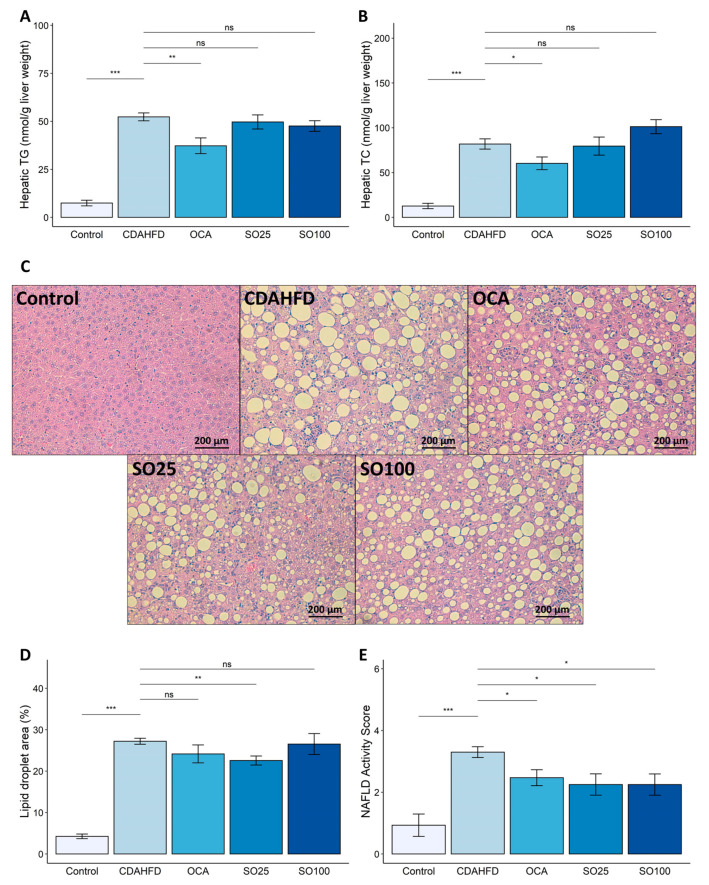
Effects of SOEE on NAFLD-related traits in CDAHFD-fed mice. (**A**) TG contents in liver tissue. (**B**) TC contents in liver tissue. (**C**) Representative image of H&E-stained liver section. Magnification is 200×. (**D**) Quantified surface area of lipid droplets in each group using ImageJ software. (**E**) Evaluated NAFLD activity score in H&E-stained liver sections using deep-learning-based CNN. Data are expressed as mean ± SEM (*n* = 10). One-way ANOVA and Tukey’s post hoc multiple comparison test were performed for statistical analysis. *p*-value compared with the CDAHFD group. “***” *p* < 0.001, “**” *p* < 0.01, “*” *p* < 0.05, and “ns” non-significant.

To determine fat depositions in the liver, which indicates NAFLD severity, we measured the hepatic TG and TC levels. The CDAHFD significantly increased the TG and TC content in the liver. The administration of OCA significantly decreased the hepatic TG and TC levels; however, the SOEE treatment did not significantly reduce the hepatic TG and TC contents (Figure 1A,B).

Histological analysis of the liver was performed to evaluate whether the SOEE inhibited hepatic lipid accumulation in the CDAHFD-fed mouse model. H&E-stained liver sections from each group were subjected to morphological analysis (Figure 1C). To compare the level of lipid accumulation in histological analysis with that in colorimetric analysis, the lipid droplet size was quantified. In the H&E-stained liver tissues, lipid accumulation was significantly higher in the CDAHFD group than in the control group (Figure 1C). The surfaces of the lipid droplets were quantified using ImageJ software (v.1.53k, NIH, USA) and showed a significant increase in lipid accumulation in the CDAHFD group compared with that in the control group (Figure 1D). In addition, treatment with the SOEE reduced the surface area of hepatic lipid droplets; however, the diminution of lipid accumulation was not observed significantly in the SO100 group.

The NAFLD activity score (NAS) was determined using a CNN model. Increased NAS levels were observed in the CDAHFD-fed mice; however, the OCA and SOEE treatments significantly reduced the NAS levels (Figure 1E). The reduction in hepatic steatosis induced by the SOEE was confirmed via various histopathological analyses.

### 3.3. Effects of SOEE on Oxidative-Stress- and Fatty-Acid-Oxidation-Related Markers in the Liver of CDAHFD-Fed Mice

**Figure 2:**
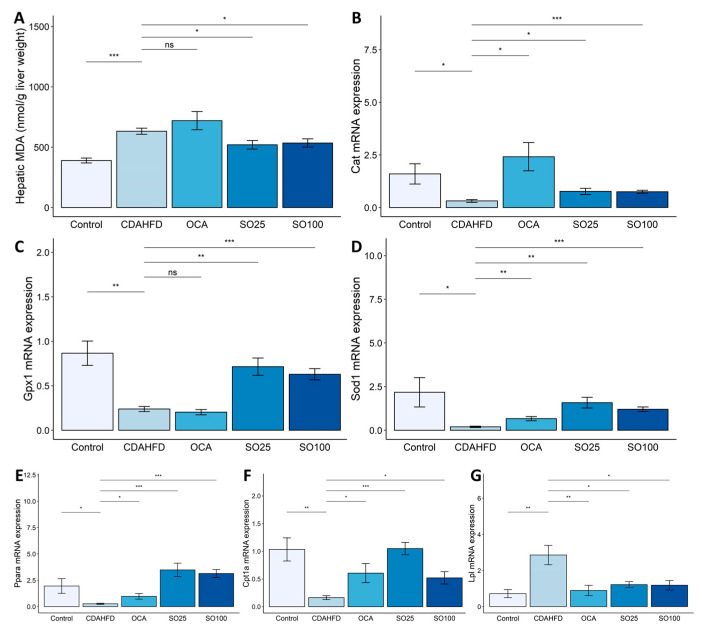
Effects of SOEE on oxidative-stress- and fatty-acid-oxidation-related markers in CDAHFD-fed mice. (**A**) Level of hepatic malondialdehyde (MDA), which is a biomarker of lipid peroxidation, measured using a colorimetric assay. (**B**,**D**) mRNA expression of oxidative-stress-related genes, namely, (**B**) catalase (*Cat*), (**C**) glutathione peroxidase I (*Gpx1*), and (**D**) superoxide dismutase 1 (*Sod1*), were quantified using qRT-PCR. mRNA expression levels of fatty-acid-oxidation-related genes were quantified using qRT-PCR. (**E**) Peroxisome proliferator-activated receptor alpha (*Ppara*), (**F**) carnitine palmitoyltransferase 1A (*Cpt1a*), and (**G**) lipoprotein lipase (*Lpl*) mRNA levels. Data are expressed as mean ± SEM (*n* = 10). One-way ANOVA and Tukey’s post hoc multiple comparison test were performed for statistical analysis. *p*-value compared with the CDAHFD group. “***” *p* < 0.001, “**” *p* < 0.01, “*” *p* < 0.05, and “ns” non-significant.

In addition to lipogenesis, multiple parallel hits involving oxidative stress and fatty acid oxidation are involved in NAFL progression [32,33,34,35]. The hepatic levels of MDA, a lipid peroxidation marker, were higher in the CDAHFD group than in the control group. The SOEE treatment significantly reduced MDA levels (*p* < 0.05) (Figure 2A). Furthermore, the expression of antioxidant enzymes, such as catalase (*Cat*), superoxide dismutase 1 (*Sod1*), and glutathione peroxidase I (*Gpx1*), which inhibit hepatic MDA production [36], was decreased in the CDAHFD group. In contrast, the SOEE treatment significantly increased the mRNA expression of antioxidant enzymes (Figure 2B–D). These results suggest that the SOEE alleviated the progression of hepatic steatosis by inhibiting oxidative stress.

PPARα target genes are involved in fatty acid oxidation in highly oxidative tissues, such as the liver, heart, and muscle [37]. Activation of PPARα induces the transcription of various fatty-acid-oxidation-related genes in the mitochondria, peroxisomes, and cytochromes, thereby reducing liver lipid levels [38,39]. *Ppara* expression was significantly lower in the CDAHFD group than in the control group (*p* < 0.05) and was recovered in the SOEE-treated group (*p* < 0.001) (Figure 2E). Moreover, mRNA expression of *Cpt1a*, one of the fatty-acid-oxidation-related genes in NASH [40], was increased in the SOEE-treated group (Figure 2F). Lipoprotein lipase (*Lpl*) plays a role in the decomposition of VLDL, which discharges TG outside the liver [41]. In this study, the OCA and SOEE treatments reduced the expression of *Lpl*, which was increased by the CDAHFD (*p* < 0.05) (Figure 2G), indicating that SOEE administration may attenuate the progression of hepatic steatosis.

### 3.4. Effects of SOEE on Hepatic Fibrosis in CDAHFD-Fed Mice

**Figure 3:**
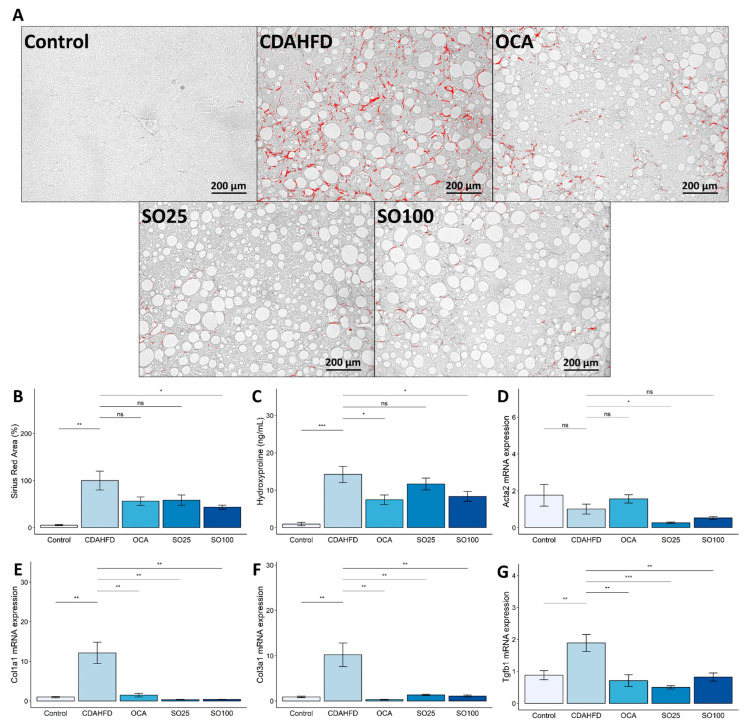
Effects of SOEE on fibrosis-related markers in the liver of CDAHFD-fed mice. (**A**) Representative image of Sirius-Red-stained liver section. Magnification was 200×. (**B**) Quantified Sirius-Red-stained area in liver tissue section (*n* = 40 images per group). (**C**) Hydroxyproline level in liver tissue (*n* = 10). (**D**–**G**) mRNA expression levels of fibrosis-related genes including (**D**) alpha-smooth muscle actin (*Acta2*), (**E**) collagen type I alpha 1 *(Col1a1)*, (**F**) collagen type III alpha 1 *(Col3a1)*, and (**G**) transforming growth factor beta 1 *(Tgfb1)* were quantified using qRT-PCR. Data are expressed as mean ± SEM (*n* = 10). One-way ANOVA and Tukey’s post hoc multiple comparison test were performed for statistical analysis. *p*-value compared with the CDAHFD group. “***” *p* < 0.001, “**” *p* < 0.01, “*” *p* < 0.05, and “ns” non-significant.

NAFLD encompasses a wide range of clinical phenotypes, including hepatic steatosis, NASH, and fibrotic NASH [42]. Histological and biochemical analyses were performed to determine the effect of the SOEE on the progression of NASH to fibrosis. Fixed liver sections were stained with Sirius Red (Figure 3A) and analyzed to quantify the fibrotic area. The Sirius-Red-stained area was significantly larger in the CDAHFD-fed mice. In contrast, the Sirius-Red-stained areas were markedly decreased (~60%) in the SO100 group (Figure 3B). In addition, the SOEE treatment inhibited liver fibrosis by reducing hydroxyproline levels, which increased with the progression of liver fibrosis (Figure 3C) [43].

The expression levels of various fibrosis-related genes were measured to estimate the occurrence of liver fibrosis. Alpha-smooth muscle actin (*Acta2*) mRNA expression was not significantly increased by the CDAHFD; however, the SO25 treatment reduced (*p* < 0.05) *Acta2* expression (Figure 3D). In addition, the expression of collagen type I alpha 1 (*Col1a1*), collagen type III alpha 1 (*Col3a1*), and transforming growth factor beta 1 (*Tgfb1*) was significantly reduced (*p* < 0.01) in the OCA, SO25, and SO100 groups compared with the CDAHFD group (Figure 3E–G). These results suggest that the SOEE treatment attenuated fibrosis progression by effectively inhibiting the CDAHFD-induced expression of fibrosis-related genes.

### 3.5. Effects of SOEE on Fibrosis-Related Gene Expression and Signaling Pathway in LX-2 Cells

**Figure 4:**
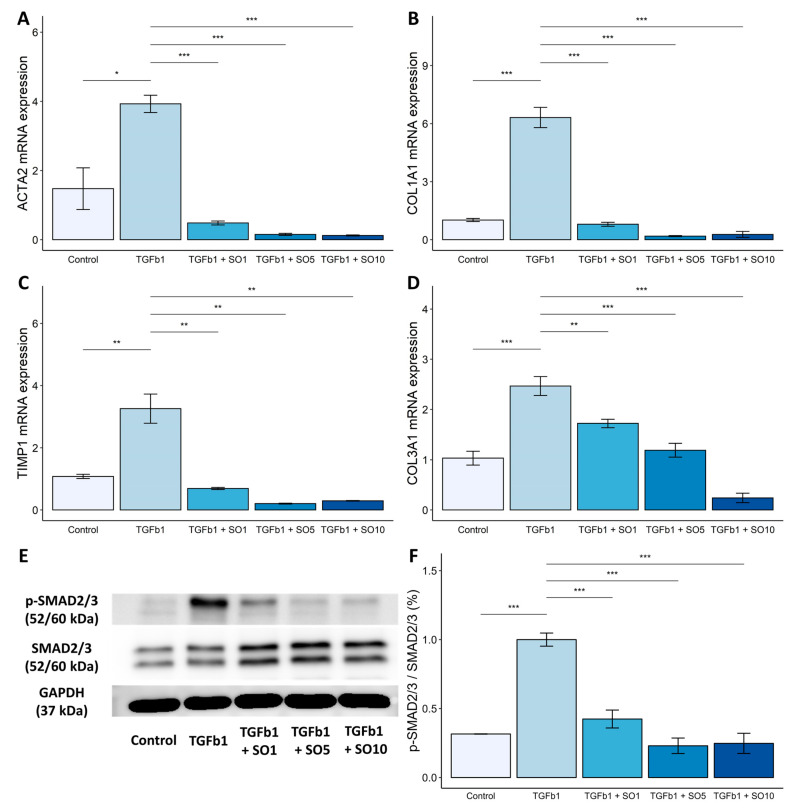
Effects of SOEE on mRNA expression levels of fibrosis-related genes and SMAD2/3 phosphorylation in LX-2 cells. To investigate the fibrosis inhibitory effect of SOEE, LX-2 cells were treated with TGF-β1 (TGFb1) at 5 ng/mL, and mRNA expression levels of (**A**) alpha-smooth muscle actin (ACTA2), (**B**) collagen type I alpha 1 (COL1A1), (**C**) TIMP metallopeptidase inhibitor 1 (TIMP1), and (**D**) collagen type III alpha 1 (COL3A1) were observed using qRT-PCR. (**E**) Protein levels of SMAD2/3 and p-SMAD2/3 were determined using Western blotting. Cells were pre-treated with SOEE for 24 h and then with TGF-β1 (5 ng/mL) and SOEE simultaneously for 30 min. (**F**) Protein expression levels were quantified using ImageJ software. Data are expressed as mean ± SEM. (*n* = 4). One-way ANOVA and Tukey’s post hoc multiple comparison test were performed for statistical analysis. *p*-value compared with the TGF-β1 group. “***” *p* < 0.001, “**” *p* < 0.01, “*” *p* < 0.05.

Next, we investigated the mechanism by which SOEE inhibited liver fibrosis in TGF-β1-induced LX-2 hepatic stellate cells (HSCs). LX-2 cells were treated with 5 ng/mL of TGF-β1 and the SOEE, and qRT-PCR was performed to confirm the inhibitory effect of the SOEE on fibrosis. First, LX-2 cells were treated with various concentrations of the SOEE (1, 5, or 10 μg/mL) in the presence of TGF-β1 (5 ng/mL). The SOEE significantly reduced the mRNA expression levels of fibrosis-related genes (*ACTA2*, *COL1A1*, *COL3A1*, and *TIMP1*), which were increased by the TGF-β1 treatment (*p* < 0.01) (Figure 4A–D). These results suggest that the SOEE had a fibrosis-inhibitory effect on HSCs and alleviated hepatic steatosis and fibrosis progression. TGF-β is a profibrogenic cytokine that primarily activates the SMAD signaling pathway [44,45]. Therefore, we hypothesized that SOEE inhibits the TGF-β signaling pathway during HSC activation and fibrosis. To determine the inhibitory effect of the SOEE on the SMAD signaling pathway, the phosphorylated form of SMAD was confirmed. Phosphorylation of SMAD2/3 was highly increased in the TGF-β1 treatment group, and the SOEE treatment significantly reduced the phosphorylation (Figure 4E,F). These results suggest that the SOEE inhibited liver fibrosis by inhibiting the phosphorylation of the key transcription factor SMAD2/3 in HSCs.

### 3.6. Effects of SOEE on the Diversity and Composition of the Gut Microbiota

**Figure 5:**
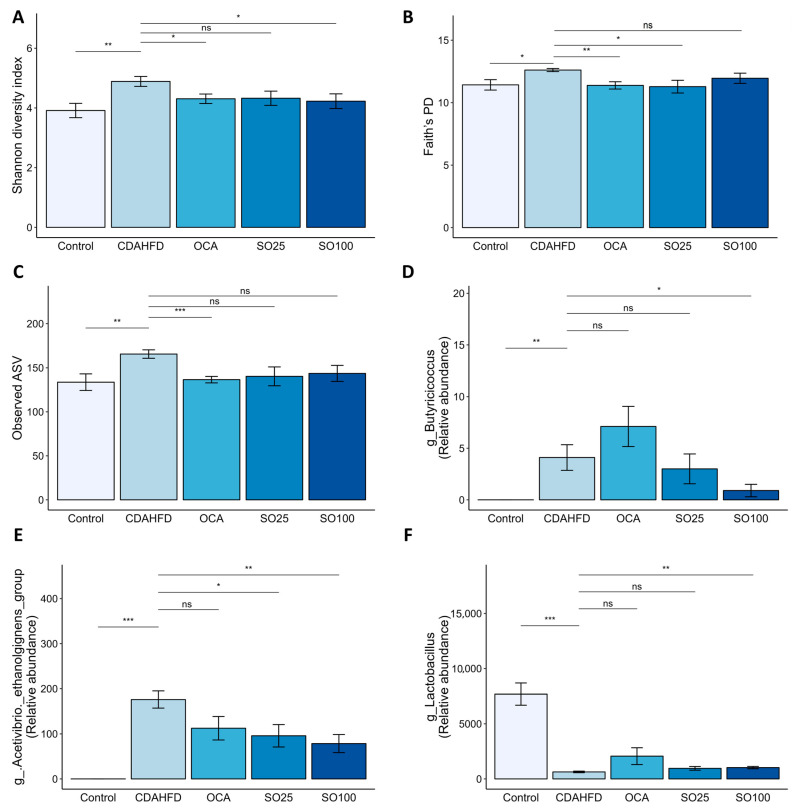

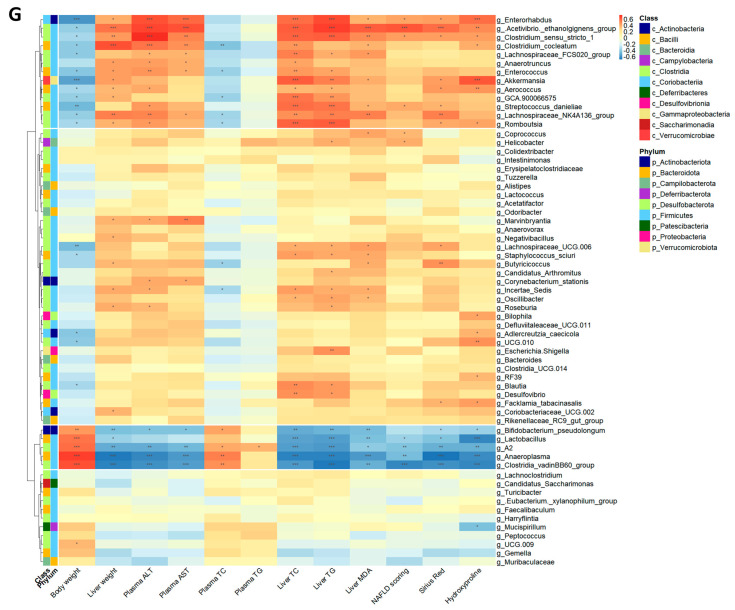
Effect of SOEE on gut microbial diversity and composition in CDAHFD-fed mice. (**A**–**C**) Alpha diversity indices: (**A**) Shannon diversity, (**B**) Faith’s PD, and (**C**) observed ASV. One-way ANOVA and Tukey’s post hoc multiple comparison test were performed for statistical analysis. *p*-value compared with the CDAHFD group. (**D**–**F**) Representative microbial genera, namely, (**D**) *Butyricicoccus*, (**E**) *Acetivibrio ethanoligignens* group, and (**F**) *Lactobacillus* show significantly differential abundance between the SOEE and CDAHFD groups. One-way ANOVA and Tukey’s post hoc multiple comparison test were performed for statistical analysis. *p*-value compared with the CDAHFD group. (**G**) Heat map showing the correlations between the abundance of microbial genera and NAFLD/fibrosis-related markers. In taxonomic classification, the class and phylum level to which each genus belongs are denoted with different colors. *p*-values were adjusted using the Benjamini–Hochberg (BH) FDR procedure. *p*-value compared with the CDAHFD group. “***” *p* < 0.001, “**” *p* < 0.01, “*” *p* < 0.05, and “ns” non-significant.

Increasing evidence suggests that the gut microbiota–liver axis plays a significant role in NAFLD/NASH, particularly in cases of fibrosis [46,47]. The 16S rRNA gene amplicon sequencing analysis of mouse fecal samples allowed us to evaluate whether the ameliorative effect of the SOEE was related to gut microbiota modulation. Analysis of the microbial diversity and differential abundance of taxa demonstrated that the CDAHFD influenced the composition and diversity of the gut microbiome. The alpha diversity index based on the Shannon metric, Faith’s PD, and the observed amplicon sequence variant (ASV) show that diversity increased in the CDAHFD group, whereas it was significantly reduced by the SOEE or OCA treatment (Figure 5A–C). To determine the microbial genera showing differential abundances between the CDAHFD and SOEE groups, we compared the microbial compositions of the two groups. The ANOVA results reveal that several bacteria, including *Butyricicoccus*, *Acetivibrio ethanolgignens*, and *Lactobacillus*, were differentially abundant between the control and CDAHFD groups (Figure 5D–F); the abundance of these genera was significantly altered by the SOEE treatment. Furthermore, the abundances of *Butyricicoccus* and *Acetivibrio ethanolgignens* were lower in the SOEE group than in the CDAHFD group and were positively correlated with NAFLD/fibrosis-related traits, whereas the abundance of *Lactobacillus* was increased in the SOEE group and was negatively correlated with NAFLD/fibrosis-related traits (Figure 5G).

## 4. Discussion

Previously reported candidates for the treatment of NAFLD, namely, OCA, elafibranor (GFT505), and lanifibranor (IVA337), were shown to improve some features of NAFLD. However, all candidates affected only a fraction of the “multiple hit” characteristic of NAFLD [48,49,50]. Candidate drugs consist of only one or two complex compounds. In contrast, natural products contain various active compounds that are effective in controlling multiple hits, which are beneficial for complex diseases. Therefore, studying the therapeutic effects of natural products and their mechanisms of action in NAFLD may be a useful strategy for the discovery of NAFLD therapeutics. SOEE was selected as a candidate compound for NAFLD inhibition using a plant extract library. To the best of our knowledge, this is the first report of SOEE as a potential candidate for NAFLD treatment, including the inhibition of fibrosis.

Many animal models have been developed using various diets [9]. Diet-induced NAFLD models, including MCD-, HFHF-, HFFC-, and CDAA-diet-induced models, were reported in various studies. Each model has disadvantages, such as severe weight loss. To effectively induce NAFLD and fibrosis and mimic human physiology, we used a CDAHFD-induced NAFLD model in this study. The effects of SOEE on NAFLD were assessed in a CDAHFD-fed mouse model and in vitro using HSCs. Several conclusions were drawn from this study. SOEE inhibited NAFLD by modulating biomarkers related to oxidative stress and fatty acid oxidation in CDAHFD-fed mice. In addition, SOEE suppressed the expression of fibrosis-related biomarkers in CDAHFD-fed mice and inhibited HSC activation by regulating SMAD signaling. Third, SOEE altered the gut microbial diversity and modulated the abundance of bacterial genera correlated with hepatic steatosis and fibrosis.

Previously, the pathogenesis of NAFLD was proposed as a “two-hit” process [51]. Recently the “multiple parallel hit” hypothesis by complex factors was reported. This hypothesis suggests that various factors, such as steatosis, the microbiome, fatty acid oxidation, and endoplasmic reticulum stress, influence the pathogenesis of NASH [32,33,34,35]. Hepatic lipid peroxidation is also associated with NAFLD [52]. MDA is a representative marker of oxidative stress and is formed via the degeneration of polyunsaturated lipids [53]. In our study, hepatic lipid accumulation occurred in the CDAHFD group, and the quantification of lipid droplets in H&E-stained liver section slides and calculation of NAS using a CNN model confirmed the reduction in steatosis using the SOEE. This analysis was particularly significant because we used a CNN to objectively assess NAS and confirm that the SOEE administration reduced hepatic steatosis. Moreover, hepatic MDA levels were significantly reduced in SOEE treatment groups. CAT, SOD, and GPX1 play crucial roles as antioxidants against reactive oxygen species [36]. The effects of CAT were determined in a *Cat^-/-^* mouse model [54]. The CDAHFD reduced the mRNA expression of *Gpx1*, *Sod*, and *Cat*, whereas the SOEE treatment increased their expression compared with the control levels. Furthermore, the SOEE increased the *Ppara* levels and suppressed *Lpl* expression in the liver. Increased LPL production prevents TG release from the liver [55]. In addition, PPARα regulates fatty acid transport and mitochondrial β-oxidation [56]. The influence of PPARα on inflammation and hepatic steatosis was demonstrated in a PPAR^-/-^ mouse model [57]. As these biomarkers are associated with fatty acid oxidation and oxidative stress, SOEE treatment may inhibit NAFLD progression by regulating fatty acid oxidation and oxidative stress.

NAFLD is a chronic inflammatory condition in which HSCs are transformed into myofibroblasts. The activation of HSCs is the most important factor in hepatic fibrosis [58]. Liver fibrosis progresses owing to HSC activation [59]. HSCs play important roles in liver physiology and fibrogenesis [60]. HSC activation leads to the excessive precipitation of the extracellular matrix (ECM) collagen [61]. ECM precipitation is consistent with the increase in hydroxyproline levels [62]. Moreover, collagen is a structural ECM protein, and its deposition results in increased mRNA levels of fibrosis-related genes, including *Col1a1,* and *Col3al* [63]. Liver fibrosis was dramatically increased in the CDAHFD-fed groups, as confirmed by fibrosis-related gene expression and histopathological analyses. The SOEE treatment significantly reduced the area of fibrosis. The level of hydroxyproline, which is an indicator of fibrosis severity, also decreased in the SO100 group. Furthermore, the SOEE significantly reduced the mRNA levels of liver-fibrosis-related genes, which were increased by the CDAHFD. Thus, the SOEE suppressed HSC activation and prevented ECM and collagen deposition.

To investigate the mechanism by which SOEE inhibits fibrosis in HSCs, liver fibrosis-related mechanisms and mRNA levels were examined in LX-2 cells. HSC activation is induced by various factors, including signaling pathway regulation, metabolic regulation, epigenetic regulation, and extracellular stimuli. TGF-β is typically the most potent factor in liver fibrosis [44,45]. For investigating the activation of HSCs, LX-2 cells were treated with TGF-β1. qRT-PCR revealed the anti-fibrotic effects of the SOEE. The SOEE markedly decreased the fibrosis-related mRNA expression in TGF-β1-treated LX-2 cells. In particular, the mRNA expression of *COL1A1* was dramatically suppressed by the SOEE. TGF-β receptor binding to TGF-β is known to induce the phosphorylation of SMAD2/3 proteins. During HSC activation, SMAD2/3 promotes the transcription of collagen types 1 and 3 [64,65]. The SOEE inhibited SMAD2/3 phosphorylation in TGF-β1-induced LX-2 cells. Natural products inhibit liver fibrosis by inhibiting SMAD2/3 [66,67]. In this study, SOEE effectively inhibited the phosphorylation of SMAD2/3 in HSCs to reduce liver fibrosis.

The SOEE increased the abundance of probiotics, such as *Lactobacillus*, which was negatively correlated with hepatic-steatosis- and fibrosis-related markers. *Lactobacillus* is a genus of lactic-acid-producing bacteria that has positive effects on gut microbiota and host health. In a clinical trial, probiotic agents, including *Lactobacillus acidophilus, Lactobacillus rhamnosus*, and *Lactobacillus paracasei*, significantly improved body weight and intrahepatic fat in patients with NAFLD [68]. In mouse models, *L. rhamnosus*, *Lactobacillus sakei*, *L. acidophilus*, *Lactobacillus fermentum*, and *Lactobacillus plantarum* significantly reduce hepatic lipid accumulation, inflammation, and plasma lipid profiles in a high-fat-diet-induced mouse model of NAFLD [69,70,71,72]. *Lactobacillus casei* supplementation reduces liver inflammation, fibrosis, and oxidative stress in rats with MCD-induced NASH [73]. *Lactobacillus* may modulate gut microbiota, reduce endotoxin levels, decrease inflammation, and improve gut barrier function, which may contribute to its therapeutic effects against NAFLD, NASH, and liver fibrosis. Therefore, SOEE combined with *Lactobacillus* spp., as a dietary supplement, may be a promising therapeutic strategy for the treatment of hepatic steatosis and fibrosis. In addition, CDAHFD mice showed an increase in the relative abundance of harmful bacteria at the genus level, including *Acetivibrio ethanolgignens*, *Roseburia*, *Rikenellaceae RC9* gut group, and *Butyricicoccus*, compared with the control group, and all of these bacteria were significantly reduced after the SOEE treatment. Furthermore, *A. ethanolgignens*, *Roseburia*, and *Butyricicoccus* were positively correlated with hepatic-steatosis- and fibrosis-related markers. *A. ethanolgignens* mediates liver inflammation and causes abnormal lipid metabolism [74]. *Roseburia*, which is a member of the *Lachnospiraceae* family, is abundant in patients with NAFLD [75]. The interaction between the gut microbiota and host is complex and variable, and SOEE can increase the relative abundance of beneficial bacteria and reduce the abundance of harmful bacteria, thereby inhibiting the development of hepatic steatosis and fibrosis.

## 5. Conclusions

In conclusion, hepatic steatosis and fibrosis were observed in the CDAHFD-induced NAFLD mouse model, and the SOEE improved oxidative stress and fatty-acid-oxidation-related factors. The SOEE markedly decreased the expression of fibrosis-related markers and inhibited the phosphorylation of SMAD2/3. Finally, we showed that the SOEE altered gut microbial diversity and composition, which are associated with hepatic steatosis and fibrosis. Thus, the SOEE, which inhibits hepatic steatosis and fibrosis by modulating oxidative stress, fatty acid oxidation, and gut microbiota, may be a potential natural therapeutic agent against NAFLD.

## Figures and Tables

**Table 1:** Primer sequence for qRT-PCR.

Genes	Direction	Primer (5′→3′)	Species
*Col1a1*	Forward	AGC ACG TCT GGT TTG GAG AG	Mouse
*Col1a1*	Reverse	GAC ATT AGG CGC AGG AAG GT	Mouse
*Col3a1*	Forward	GTG GAC ATT GGC CCT GTT TG	Mouse
*Col3a1*	Reverse	AGT TGG TCA CTT GCA CTG GT	Mouse
*Tgfb1*	Forward	GTG GCT GAA CCA AGG AGA CG	Mouse
*Tgfb1*	Reverse	GTT TGG GGC TGA TCC CGT TG	Mouse
*Timp1*	Forward	TTA TTC TCC ACT GTG CAG CCC	Mouse
*Timp1*	Reverse	ACA AGA GGA TGC CAG ATG CC	Mouse
*Acta2*	Forward	TCC AGC CAT CTT TCA TTG GGA	Mouse
*Acta2*	Reverse	CCC CTG ACA GGA CGT TGT TA	Mouse
*Ppara*	Forward	GAA CTG ACG TTT GTG GCT GG	Mouse
*Ppara*	Reverse	GCT CTC TGT GTC CAC CAT GT	Mouse
*Cpt1a*	Forward	ACT CCG CTC GCT CAT TCC	Mouse
*Cpt1a*	Reverse	GAC TGT GAA CTG GAA GGC CA	Mouse
*Lpl*	Forward	GTG GAC ATC GGA GAA CTG CT	Mouse
*Lpl*	Reverse	CCT CTC GAT GAC GAA GCT GG	Mouse
*Sod1*	Forward	GGG AAG CAT GGC GAT GAA AG	Mouse
*Sod1*	Reverse	GCC TTC TGC TCG AAG TGG AT	Mouse
*Cat*	Forward	CAA GAT TGC CTT CTC CGG GT	Mouse
*Cat*	Reverse	ATG GTG TAG GAT TGC GGA GC	Mouse
*Gpx1*	Forward	AGT CCA CCG TGT ATG CCT TC	Mouse
*Gpx1*	Reverse	CCT CAG AGA GAC GCG ACA TT	Mouse
*β-actin*	Forward	CAT TGC TGA CAG GAT GCA GAA GG	Mouse
*β-actin*	Reverse	TGC TGG AAG GTG GAC AGT GAG G	Mouse
*ACTA2*	Forward	GCC AAG CAC TGT CAG GAA	Human
*ACTA2*	Reverse	ATT GTC ACA CAC CAA GGC A	Human
*COL1A1*	Forward	ATG GAG CTC CTG GTC AGA T	Human
*COL1A1*	Reverse	GTA GCA CCA TCA TTT CCA CG	Human
*COL3A1*	Forward	GCT CTG CTT CAT CCC ACT AT	Human
*COL3A1*	Reverse	CGC ATA GGA CTG ACC AAG AT	Human
*TIMP1*	Forward	CTC TGA AAA GGG CTT CCA GTC	Human
*TIMP1*	Reverse	AGG ATT CAG GCT ATC TGG GAC	Human
*GAPDH*	Forward	CAG CCG CAT CTT CTT TTG CG	Human
*GAPDH*	Reverse	TCC GTT GAC TCC GAC CTT CA	Human

**Table 2:** Effects of SOEE on plasma lipid profiling and liver injury markers in CDAHFD-fed mice.

Group	Body Weight (g)	Liver Weight (g)	ALT (IU/L)	AST (IU/L)	Plasma TG (mM/L)	Plasma TC (mM/L)
Control	*** 30.87 ± 3.38	*** 1.15 ± 0.18	*** 1.12 ± 0.56	*** 5.49 ± 2.09	0.36 ± 0.11	* 0.99 ± 0.14
CDAHFD	24.16 ± 2.34	1.67 ± 0.26	17.85 ± 2.58	13.13 ± 2.50	0.40 ± 0.10	0.83 ± 0.17
OCA	23.32 ± 1.99	** 2.04 ± 0.20	^#^ 24.45 ± 10.28	* 25.69 ± 15.03	*** 0.23 ± 0.07	^#^ 0.67 ± 0.17
SO25	24.19 ± 1.81	1.68 ± 0.29	15.84 ± 4.41	^#^ 10.36 ± 3.48	* 0.30 ± 0.06	0.77 ± 0.15
SO100	24.50 ± 1.15	1.67 ± 0.19	18.24 ± 3.52	11.31 ± 3.11	** 0.27 ± 0.07	0.72 ± 0.11

Data are expressed as mean ± SEM. One-way ANOVA and Tukey’s post hoc multiple comparison tests were performed for statistical analysis. *p*-value compared with the CDAHFD group. “***” *p* < 0.001, “**” *p* < 0.01, “*” *p* < 0.05, and “^#^” *p* < 0.10.

## Data Availability

The authors confirm that all data underlying the findings are fully available and can be obtained after submitting a request to the corresponding author.

## Supplementary Materials

The following supporting information can be downloaded from https://www.mdpi.com/article/10.3390/nu15173779/s1: Figure S1. HPLC chromatograms of (A) 95% ethanol extract of *Sanguisorba officinalis* L. (SOEE) at 203 nm, (B) standard of ziyuglycoside I and II (203 nm), (C) HPLC chromatograms of SOEE at 280 nm, and (D) standard gallic acid (280 nm); Figure S2. Experimental design of CDAHFD-induced mouse model; Table S1. HPLC conditions for the analysis of SOEE and standards of ziyuglycoside I and II; Table S2. HPLC conditions for the analysis of SOEE and gallic acid standard; Table S3. Calibration data for ziyuglycoside I and II and gallic acid; Table S4. Ziyuglycoside I, ziyuglycoside II, and gallic acid content in SOEE.
