# Supplementary material for: Sanguisorba officinalis L. Ameliorates Hepatic Steatosis and Fibrosis by Modulating Oxidative Stress, Fatty Acid Oxidation, and Gut Microbiota in CDAHFD-Induced Mice
Source: Nutrients. 2023 Aug 29;15(17):3779. doi: 10.3390/nu15173779 (PMC10490207; doi:10.3390/nu15173779)
Supplement: Supplementary file 1 [file nutrients-15-03779-s001.zip › nutrients-2560022-supplementary/[nutrients-2560022]Proofreading_supplementary_materials.pdf]

Sanguisorba officinalis L. ameliorates hepatic steatosis and fibrosis by modulating oxidative stress, fatty acid oxidation, and gut microbiota in CDAHFD-induced mice

Yunseong Nam[1,2,†], Myungsuk Kim[1,3,4,†], Saruul Erdenebileg[1,2], Kwang Hyun Cha[1,4,5], Da Hye Ryu[2], Ho Youn Kim[1,2], Su Hyeon Lee[2], Je Hyeong Jung[2], and Chu Won Nho[1,2,*]

[1] Division of Bio-Medical Science and Technology, KIST School, University of Science and Technology (UST), Seoul 02792, Republic of Korea

[2] Smart Farm Research Center, Korea Institute of Science and Technology (KIST),

Gangneung 25451, Republic of Korea

[3] Natural Product Research Center, Korea Institute of Science and Technology (KIST),

Gangneung 25451, Republic of Korea

[4] Department of Convergence Medicine, Wonju College of Medicine, Yonsei University,

Wonju 26493, Republic of Korea

[5] Natural Product Informatics Research Center, Korea Institute of Science and Technology (KIST),

Gangneung 25451, Republic of Korea

* Correspondence: cwnho@kist.re.kr; Tel.: +82-(33)-650-3420

† These authors contributed equally to this work.

Supplementary materials and methods

1. *HPLC analysis*

A high-performance liquid chromatograph (HPLC) (Agilent 1260 series, USA) equipped with a binary pump, autoinjector, column thermostat, and diode array detector (DAD) was used for HPLC analysis with minor modifications. Ten microliters of injected samples and standards were separated on a YMC C18 Triart column (250×4 mm, 5 μm) maintained at 35°C, and both solvents were acidified using 0.2% formic acid as mobile phase (A: water containing 0.2% formic acid B: acetonitrile containing 0.2% formic acid). The flow rate was kept at 0.8 mL per minute and a gradient elution program was used. A gradient system was used for compound detection. The initial B solvent was 0%, and was maintained for 5 min, and then changed to 10% B for 10 min, 10% B for 15 min, 15% B for 20 min, 15% B for 25 min, 17% B for 30 min, 17% B for 35 min. The percentage of B solvent increased rapidly to 25% at 45 min, 50% at 55 min, and 100% at 65 min. This condition (100% B) was maintained for 2 min and a post-run was applied for 5 min. Analog out channel A at wavelength 280 nm for gallic acid quantification and analog out channel B at 203 nm for ziyuglycoside I and II both with bandwidth 4 nm.

2. *Quantification of compounds in 95% ethanol Sanguisorba officinalis* L. *extract (10 mg/mL)*

Standard of gallic acid (Cat. CFN99624), ziyuglycoside I (Cat. CFN98464), and ziyuglycoside II (Cat. CFN984653) were obtained from ChemFaces (Wuhan, China). Quantification was conducted by the integration of the peak using external standard method. The plots were drawn in concentration range of 0.025 to 1.0 mM for gallic acid (Rt: 12.554 min) and 0.25 to 5.0 mM for ziyuglycoside I (Rt: 55.375 min) and ziyuglycoside II (Rt: 61.471 min) quantification with good regression characteristics (correlation coefficient (R2) > 0.995). And the linear regression equations were as follows:

Gallic acid: Y = 36.339X – 5.1561 (R2 = 0.9988)

Ziyuglycoside I: Y = 2.3998X – 549.19 (R2 = 0.9952)

Ziyuglycoside II: Y = 1.1785X – 51.459 (R2 = 0.9990),

Y means the peak area and X indicates content of each compound

3. *Histological examination*

The right lobe of the liver from each mouse was fixed in 4% paraformaldehyde. The fixed liver tissues were embedded in paraffin. Formalin-fixed liver sections were stained with hematoxylin and eosin (H&E) and Sirius red. Liver images were obtained using a Zeiss AxioCam MRc5 microscope (Carl Zeiss, Jena, Germany). H&E- and Sirius red-stained images were obtained from ten slides in each group, with four images per slide. ImageJ software (National Institutes of Health, Bethesda, MD, USA) was used to quantify and analyze the stained areas.

4. *Quantitative reverse transcription-polymerase chain reaction (qRT-PCR)*

Total RNA was extracted from liver tissue and cells using a HybridR™ RNA isolation kit (GeneAll, Seoul, Korea), and complementary DNA (cDNA) was synthesized using the PrimeScript™ 1st strand cDNA Synthesis Kit (Takara, Kyoto, Japan). qRT-PCR was performed using PowerUp™ SYBR™ Green Master Mix (Thermo Fisher Scientific, Waltham, MA, USA) and gene-specific primers (Table 1). A Light Cycler 480 Real-Time PCR System (Roche, Basel, Switzerland) was used to analyze the mRNA

expression levels. Target mRNA expression were normalized by housekeeping gene expression (β-actin and GAPDH).

5. *Western blotting*

Cell and tissue lysates were harvested using radioimmunoprecipitation assay (RIPA) buffer (Thermo Fisher Scientific) containing a protease inhibitor cocktail and phenylmethylsulfonyl fluoride (Sigma). After cell lysis, the lysate was centrifuged at 13000 rpm for 30 min to obtain proteins. The proteins were separated and transferred onto polyvinylidene fluoride (PVDF) membranes (Bio-Rad, Hercules, CA, USA). After blocking the membranes with 3% bovine serum albumin for 1 h in phosphate-buffered saline-Tween 20 (PBST), they were incubated with specific primary and secondary antibodies. The primary antibodies used were anti-rabbit SMAD2/3 (cat. no.5678; Cell Signaling Technology (CST), Danvers, MA, USA), phosphor-SMAD2/3 (Cat. 8828, CST, USA), and glyceraldehyde-3-phosphate dehydrogenase (GAPDH) (Cat. sc-25778, Santa Cruz Biotechnology, Dallas, TX, USA). Goat anti-rabbit IgG antibody (horseradish peroxidase (HRP)-conjugated) (cat. GTX213110-01; GeneTex, Irvine, CA, USA) was used as the secondary antibody. Proteins were developed using the SuperSignal™ reagent (Thermo Fisher Scientific, USA). The protein bands were detected using LAS-4000 (Fujifilm, Tokyo, Japan) and quantified using ImageJ software (NIH, USA).

6. *16S rRNA gene sequencing of the bacterial community in feces*

Fecal samples were homogenized by mechanical lysis using bead beating, and DNA was extracted using the QIAamp® Fast DNA Stool Mini Kit (Qiagen, Hilden, Germany) according to the manufacturer's instructions. To amplify the V3–V4 hypervariable region of the 16S rRNA gene, PCR was performed using the universal primer set 341F and barcoded 806R. Denaturing gradient gel electrophoresis was used to confirm the PCR products, and the gel was purified using AMPure XT beads (Beckman Coulter Genomics, Danvers, MA, USA) and Qubit dsDNA High-Sensitivity Reagent (Invitrogen, Carlsbad, CA, USA). Samples were sequenced on a MiSeq platform using a paired-end 2 × 300-bp reagent kit (Illumina, San Diego, CA, USA), and raw sequencing data were analyzed using the QIIME2-DADA2 pipeline[1,2] to determine amplicon sequence variants (ASV). Taxonomic classification was performed at the phylum, class, order, family, and genus levels and generated using QIIME 2 software. For alpha diversity (Shannon diversity index, Faith's phylogenetic diversity, and Observed ASVs), analyses were performed using PhyloSeq to import the data generated from QIIME2 into R (v.4.1.2) (R Core Team).

Table S1. HPLC conditions for the analysis of SOEE and standards of ziyuglycoside I and II.

HPLC condition	Condition 1		
Column	YMC C18 Triart column		
Column temp.	35		
Flow rate	0.8 mL/min		
Wave length	203 nm		
Injection volume	10 μL		
Mobile Solvent	A: water containing 0.2% formic acid<br>B: Acetonitrile containing 0.2% formic acid		
Mobile phase	Time (min)	A (%)	B (%)
	0	100	0
	5	100	0
	10	90	10
	15	90	10
	20	85	15
	25	85	15
	30	83	17
	35	83	17
	45	75	25
	55	50	50
	65	0	100
	67	0	100

Table S2. HPLC conditions for the analysis of SOEE and gallic acid standard.

HPLC condition	Condition 2		
Column	YMC C18 Triart column		
Column temp.	35		
Flow rate	0.8 mL/min		
Wave length	280 nm		
Injection volume	10 μL		
Mobile Solvent	A: water containing 0.2% formic acid<br>B: Acetonitrile containing 0.2% formic acid		
Mobile phase	Time (min)	A (%)	B (%)
	0	100	0
	5	100	0
	10	90	10
	15	90	10
	20	85	15
	25	85	15
	30	83	17
	35	83	17
	45	75	25
	55	50	50
	65	0	100
	67	0	100

Table S3. Calibration data for ziyuglycoside I and II, and gallic acid.

Compound	Linear range (mM)	Response Slope (a)	Response Factor (b)	Correlation coefficient (R^2)	CV (%)	LOD (mM)	LOQ (mM)
Gallic acid	0.025 ~ 1.0	36.339	-5.1561	0.9988	0.17	0.0003	0.0009
Ziyuglycoside I	0.25 ~ 5.0	2.3998	549319	0.9952	0.02	0.484	1.467
Ziyuglycoside II	0.25 ~ 5.0	1.1785	51.459	0.9990	0.02	0.442	1.340

Table S4. Ziyuglycoside I, ziyuglycoside II, and gallic acid content in SOEE.

	Contents of compounds in SOEE		
	Gallic acid	Ziyuglycoside I	Ziyuglycoside II
Rt	12.554	55.375	61.474
Peak area	3772.7	2979.8	1883
Content (mg/g)	10.40	101.28	155.41

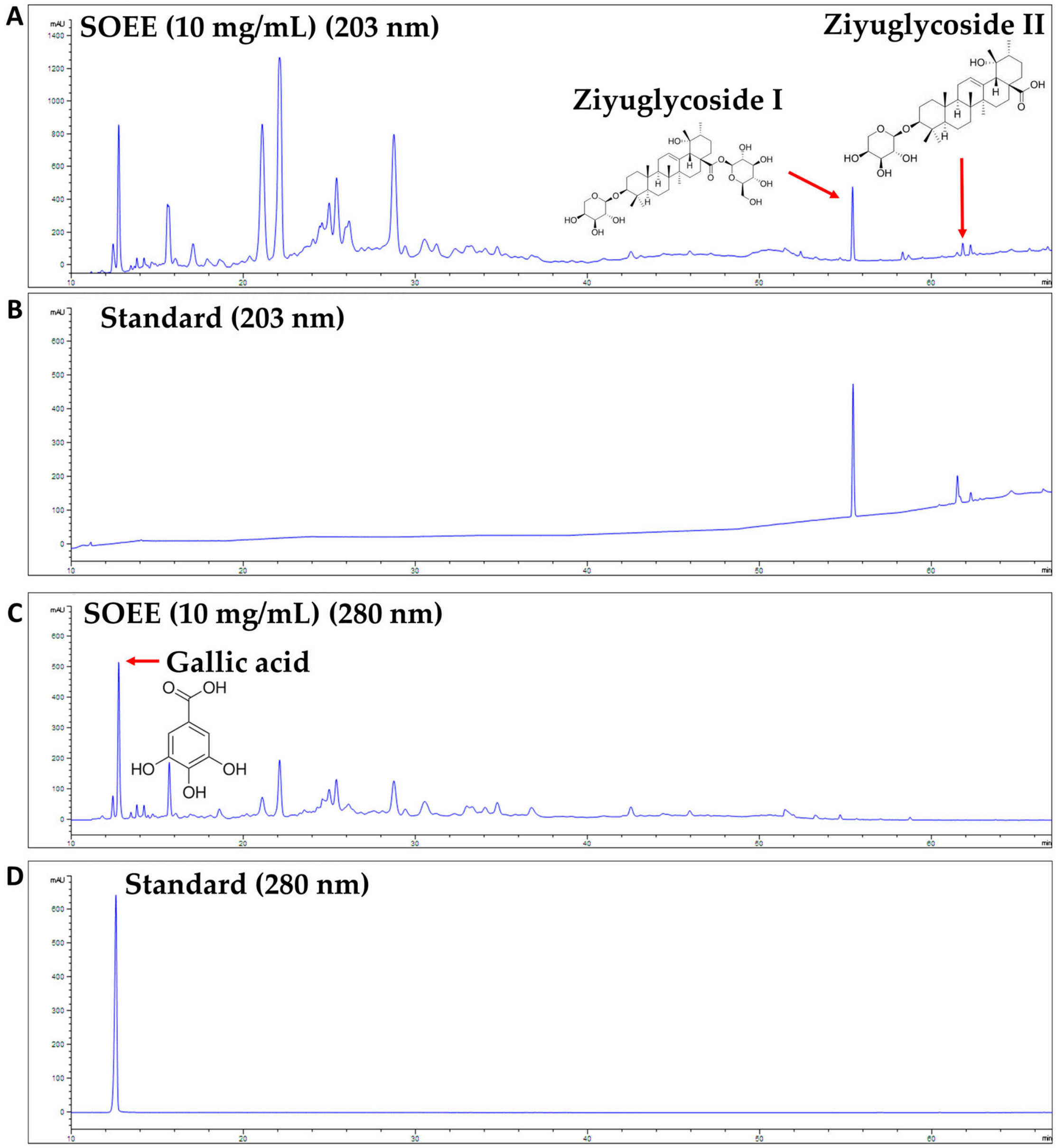

Figure S1. HPLC chromatograms of (A) 95% ethanol extract of *Sanguisorba officinalis* L. (SOEE) at 203 nm, (B) standard of ziyuglycoside I and II (203 nm), (C) HPLC chromatograms of SOEE at 280 nm, and (D) standard gallic acid (280 nm).

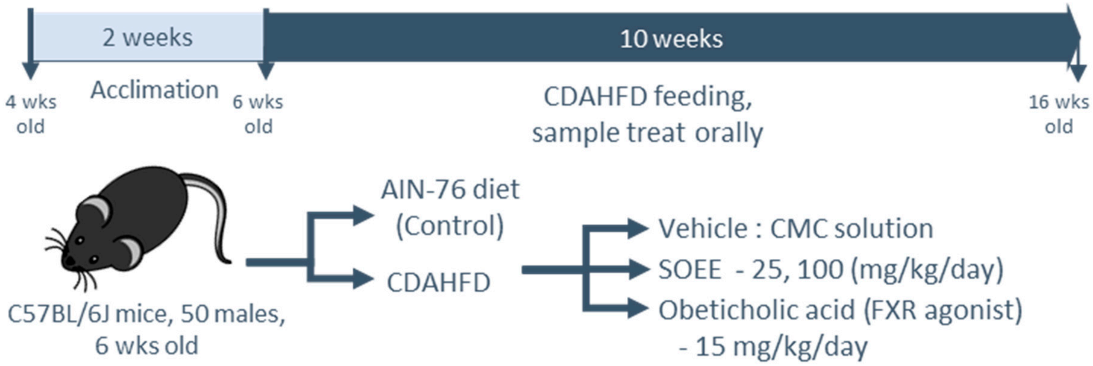

Figure S2. Experimental design of CDAHFD-induced mouse model.

Reference

1. Bolyen, E.; Rideout, J.R.; Dillon, M.R.; Bokulich, N.A.; Abnet, C.C.; Al-Ghalith, G.A.; Alexander, H.; Alm, E.J.; Arumugam, M.; Asnicar, F. Reproducible, interactive, scalable and extensible microbiome data science using QIIME 2. *Nature biotechnology* **2019**, *37*, 852-857.
2. Callahan, B.J.; McMurdie, P.J.; Rosen, M.J.; Han, A.W.; Johnson, A.J.; Holmes, S.P. DADA2: High-resolution sample inference from Illumina amplicon data. *Nat Methods* **2016**, *13*, 581-583, doi:10.1038/nmeth.3869.
